# LIMPRINT: The UK Experience—Subjective Control of Swelling in Patients Attending Specialist Lymphedema Services

**DOI:** 10.1089/lrb.2019.0020

**Published:** 2019-04-22

**Authors:** Christine J. Moffatt, Vaughan Keeley, Andrew Hughes, Kath Clark, Jill Lisle, Margaret Benson, Rebecca Gaskin, Martina Sykorova, Eleanor Dring, Susie Murray, Gregoire Mercier, Isabelle Quéré, Peter J. Franks

**Affiliations:** ^1^ School of Social Sciences, Nottingham Trent University, Nottingham, United Kingdom.; ^2^ Montpellier Medecine Vasculaire, EA2992, Universite Montpellier I, CHU Saint Eloi, Montpellier, France.; ^3^ Copenhagen Wound Healing and Lymphoedema Centre, Bisperberg University Hospital, Copenhagen, Denmark.; ^4^ Lymphoedema Service, Royal Derby Hospital, Derby, United Kingdom.; ^5^ St Oswalds Hospice, Newcastle Upon Tyne, United Kingdom.; ^6^ LOROS, Leicester, United Kingdom.; ^7^ Royal Derby Hospital Centre, School of Health Sciences, University of Nottingham, Derby, United Kingdom.; ^8^ Queens Medical Centre, School of Health Sciences, University of Nottingham, Nottingham, United Kingdom.; ^9^ Centre for Research & Implementation of Clinical Practice, London, United Kingdom.

**Keywords:** control of swelling, chronic edema, arm, leg, risk factors, lymphedema, lymphoedema

## Abstract

***Background and Study Design:*** This study was undertaken as part of the UK LIMPRINT international study to determine the number of people with chronic edema (CO) and its impact on health services. Overall 7436 with CO were recruited in the main UK study from a range of health settings.

***Methods and Results:*** Factors relating to subjective control of arm and leg CO were defined in the UK. A total of 1565 patients were included in the study with exclusions for: no limb swelling or not recorded (1669), having concurrent arm/leg CO (272), control of assessment missing (5) and professional being unsure of control status of CO (325). Arm swelling occurred in 953 (18.5%) with leg CO in 4212 (81.5%). Poor control was found in 1430 (27.2%) and good control in 3735 (72.3%). Control of arm swelling was worse in men and control increased overall in those aged over 45 years. In contrast control of CO worsened in those with leg CO with increasing age and multiple co-morbidities. Obesity and cellulitis, particularly an episode in the last year were associated with poor control. Independent risk factors for arm CO were : obesity, neurological disease and cellulitis in the last year and for leg CO, obesity, poor mobility, heart disease, presence of a wound, cellulitis in the last year and duration of swelling.

***Conclusion:*** Control of CO within specialized centers is complex due to sociodemographic and clinical comorbidities.

## Background to Lymphedema Services in the United Kingdom

As in most countries of the world, provision of treatment for patients with Lymphedema in the United Kingdom is poor. Historically services were only offered to those suffering from Lymphedema following cancer treatment, and those with primary and other forms of chronic edema (CO) were denied access to care. This led to an increasingly biased view that Lymphedema was only a manifestation of the cancer population rather than a complex heterogenous population.

## The UK Lymphedema Framework

Nearly two decades ago funding was obtained from the Kings Fund to develop a collaborative approach to try to improve UK treatment provision. The project involved epidemiology, development of the Best Practice Document, and evaluation of a model of care. This work has been published in full.^1–4^ In 2006, the partners within this project were also successful in obtaining NHS Drug Tariff approval for Lymphedema products to be listed for prescribing in the community through general practices and community nurses. This opened the way for community-based treatment and stimulated investment from the medical device industry to develop new products and invest in this area. Despite this there is still patchy care provision across the United Kingdom with only Wales having a national strategy for care provision.^5^

Since then much effort has been provided by the National Lymphoedema Partnership to improve the national recommendations for treatment. The UK Lymphoedema Framework led to the development of the International Lymphoedema Framework, which embraces the importance of partnership working and addressing some of the key questions internationally that must be addressed.

## Development of the Definition of CO in the United Kingdom

The recognition of the complex patients presenting to a national UK specialist services two decades ago led to the development of the term “chronic oedema.”^1^ This was used in the prevalence study carried out in 2003 and is defined below:

“Chronic oedema is a broad term used to describe oedema which has been present for more than three months and involves one or more of the following areas: limbs, hands/feet, upper body (breast/chest wall, shoulder, back), lower body (buttocks, abdomen), genital (scrotum, penis, vulva), head, neck or face.”

Thus, “chronic oedema” can be considered to be an umbrella term, which includes not only conventional “Lymphoedema” but also chronic swelling which may have a more complex cause.

## Prevalence Studies in the UK

The first study to use the definition of CO was reported in 2003.^1^ The aim of the study was to determine the magnitude of the problem of CO in health services within an urban area of London, United Kingdom, and to assess the likely impact of edema on use of health resources, employment, and patient's quality of life. The study used a questionnaire-based survey given to health professionals followed by an interview and clinical assessment in a random sample. Health professionals from dedicated Lymphedema services, specific outpatient clinics, hospital wards, and community services (GP clinics and district nurses) were contacted to provide information on patients from within the geographical area who were known to suffer with CO of greater than 3 months duration.

Within the catchment area with a population of 619,000 people, 823 patients had CO (crude prevalence 1.33/1000). Prevalence increased with age (5.4/1000 in those aged >65 years) and was higher in women (2.15 vs. 0.47/1000). Only 529 (64%) were receiving treatment, despite two specialist Lymphedema clinics within the catchment area. Of 228 patients interviewed, 78% had edema lasting >1 year. Over the previous year, 64/218 (29%) had had an acute infection in the affected area, with 17/64 (27%) being admitted to hospital for intravenous antibiotics. Mean length of stay for this condition was 12 days, with an estimated mean cost of £2300 (2003 data). Edema caused time off work in >80% and affected employment status in 9%. Quality of life was below normal, with 50% experiencing pain or discomfort from their edema.

Using an extrapolation of these figures, it was estimated that at least 100,000 patients were suffering in the United Kingdom alone. However, it is acknowledged that this will be an underestimate of the true prevalence within the general population.

This methodology was repeated over 10 years later in an urban population of the East Midlands in the United Kingdom.^2^ This cross-sectional study was carried out in Derby City (United Kingdom), which has a population of ∼247,100. Data were obtained from 10 sources, namely: the inpatients of one acute and one community hospital, one specialist and three nonspecialist outpatient clinics (dermatology, plastic surgery, and diabetic foot clinic), all community nursing services, general practices (*n* = 41), and nursing/residential homes (*n* = 26) in the catchment area.

Within the study population of Derby City residents, 971 patients were identified with CO (estimated crude prevalence 3.93 per 1000, 95% CI 3.69–4.19). The prevalence was highest among those aged 85 or above (28.75 per 1000) and was higher among women (5.37 per 1000) than men (2.48 per 1000). The prevalence among hospital inpatients was 28.5%. Only 5 (3%) patients in the community population had edema related to cancer or cancer treatment. Patients with cancer related lymphedema were usually treated by hospital-based services in Derby. Of the 304 patients identified with edema from the Derby hospitals or community health services, 121 (40%) had a concurrent leg ulcer.

## The Role of Professional Judgment of Control of Swelling

Internationally agreed definitions of outcome of CO management are lacking. While limb volume measurement is frequently cited as the primary outcome of many studies, there are many variations to this technique, and a lack of standardization makes interpretation between studies difficult. Therefore, clear conceptualization of how professionals and patients define control of swelling is urgently needed.

Within the original epidemiology conducted in the United Kingdom, professionals who identified participants were asked to make a subjective assessment of whether they considered that the participants CO was well controlled.^1^ They also identified whether they were receiving treatment and the site of swelling. All personnel involved in the study were trained in the data collection methods and had access to Lymphedema experts if they were unsure whether the CO was stable. Within the sample of 823 participants health professionals considered control of swelling to be better in women compared to men (62.5% vs. 44.3%). In the youngest age group (<45 years), only 11% were reported to have uncontrolled swelling. This increased with age to 59.0% in those aged >85 years. Professionals reported that patients with arm edema experienced better control than patients with leg edema (85% vs. 42%). Nearly 80% (77.9%) of patients who were being actively treated for their CO had control, compared with just 29.4% in those not being offered treatment. Despite the subjective nature of this question it would appear that there are important associations influencing control of swelling that are not only related to the complex patient population but also to effective care provision.

## Overall Summary of LIMPRINT in the United Kingdom

The LIMPRINT study was undertaken in different health settings in the United Kingdom. The study adopted the methods and data collection tools previously defined in the LIMPRINT methodology.^6^ Results from these studies have been published separately.^7,8^ In summary, 7436 participants were recruited from the following settings: specialist Lymphedema services (*n* = 5660), community nursing services (*n* = 1440), inpatient hospital facilities (NUH trust) (*n* = 298), and other settings, including a prison (*n* = 38) (Table 1). The population was predominantly female (71.8%) with a mean age of 66.6 years. Obesity was common, affecting 37.1% with a further 20.7% defined as morbidly obese. While half could walk unaided, 12% were either chair or bedbound. Swelling affecting the leg was the most common (79.7%) with arm edema affecting 21.2% and a further 12% having midline swelling with concurrent limb swelling. The majority had secondary CO (87.4%) that was unrelated to having cancer (67.5%). A further 12.5% were defined as suffering from primary Lymphedema.

**Table 1.** Total UK LIMPRINT Study: (*N* = 7436) Demographic, Mobility, and Swelling Related Problems

	*Home care* N *(%)*	*Inpatient* N *(%)*	*Specialist service* N *(%)*	*Other* N *(%)*	*Total* N *(%)*
*N*	1440	298	5660	38	7436
Gender					
Female	842 (58.47)	163 (54.70)	4309 (76.13)	26 (68.42)	5340 (71.81)
Male	598 (41.53)	135 (45.30)	1351 (23.87)	12 (31.58)	2096 (28.19)
Age					
Mean (SD)	76.3 (13.8)	73.0 (16.4)	64.4 (15.8)	69.1 (12.0)	66.6 (16.7)
Obesity					
Under weight	132 (9.17)	27 (9.06)	64 (1.13)	5 (13.16)	228 (3.07)
Normal weight	805 (55.90)	136 (45.64)	1945 (34.37)	24 (63.16)	2910 (39.14)
Obese	408 (28.33)	102 (34.23)	2244 (39.65)	7 (18.42)	2761 (37.14)
Morbidly obese	95 (6.60)	33 (11.07)	1406 (24.85)	2 (5.26)	1536 (20.66)
Lower limb mobility					
Walks unaided	409 (28.42)	74 (24.83)	3343 (59.08)	2 (5.26)	3828 (51.50)
Walks with aid	760 (52.81)	157 (52.68)	1794 (31.71)	25 (65.79)	2736 (36.81)
Chair bound	181 (12.58)	34 (11.41)	498 (8.80)	11 (28.95)	724 (9.74)
Bedbound	89 (6.18)	33 (11.07)	23 (0.41)	0 (0)	145 (1.95)
Swelling					
Arm/hand	^*^	56 (19.05)	1210 (21.43)	5 (13.16)	1271 (21.26)
Leg/foot	^*^	279 (94.90)	4451 (78.85)	38 (100)	4768 (79.77)
Midline	^*^	31 (10.54)	685 (12.13)	3 (7.89)	719 (12.03)
Classification					
Primary	^*^	11 (3.69)	734 (13.12)	0	745 (12.57)
Secondary	^*^	287 (96.31)	4859 (86.88)	38 (100)	5184 (87.43)
Secondary					
Cancer	^*^	31 (10.80)	1646 (34.07)	8 (21.05)	1685 (32.68)
Noncancer	^*^	256 (89.20)	3185 (65.93)	30 (78.95)	3471 (67.32)

^*^ Not recorded.

## Methods to Assess Subjective Control of Swelling Within Specialist Lymphedema Services

### Setting and sampling frame

The study to investigate professional views on control of swelling was carried out in three regional Lymphedema Services in the Midlands and north of England, United Kingdom (Derby, LOROS, Leicester, and St. Oswald's Hospice, Newcastle). These services participated in the main UK LIMPRINT study. Data were obtained using the same methodology in each area. Approval for the studies was granted by the local Research and Governance Committees for each service area and complied with their regulations.

### Identification of patients through the creation of master lists

A master list was created using the data systems and clinical notes within each service and checked for duplication and deaths. The reasons for all exclusions were recorded. All forms were checked against the master list to ensure complete data capture. Individualized and anonymized participant numbers were allocated to avoid duplication of recruitment. The questionnaire and process of clarification were piloted with the team at St Oswald's Hospice.

The following information was used to collect individual data on *subjective control of swelling*:

- The current CO status was obtained from the clinical notes based on their last clinical visit and limb volume measurement
- Newly presenting patients who were not yet receiving treatment were noted
- Patients receiving complex decongestive therapy (CDT) at the time of recruitment were noted
- Patients in maintenance therapy (self-management) with skin care, exercise, self-massage, and compression hosiery were noted
- A history of cellulitis and an episode of cellulitis in the last year were noted
- Classification of control of swelling was clarified with the therapists treating the patient and where necessary with the lead physician within each service

## Statistical Methods

### Data analysis

Data were entered onto a bespoke data entry and validation system.^6^ After completion of patient entry, the data were downloaded to an Excel spreadsheet and exported into Stata 12 (StataCorp, TX), where statistical analyses were undertaken. After exclusions, patients were allocated to two groups, either those with arm swelling or those with leg swelling. Factors associated with control in these subsets were then undertaken. The primary method of analysis was logistic regression with either control or poor/no control as the dependent variable. Odds ratios (OR) and 95% confidence intervals were used to describe the difference between the independent risk categories and the dependent variable. The *p*-values were derived from the Chi squared results derived from the logistic regression. Finally, an analysis was undertaken to identify independent factors that were associated with the presence of CO in the cohort.

## Results

In total there were 7436 patients seen by health professional in the study areas of the United Kingdom, Table 1. The majority of these were seen in specialist lymphedema services (5660, 76.1%). As expected most (71.8%) were female, with a mean (SD) age of 66.6 (16.7) years. While those seen in Home Care were mainly of normal weight, a greater proportion in Specialist services were either considered to be obese or morbidly obese. As expected, those patients seen at home or as inpatients had greater mobility deficits compared with specialist services. The distribution of sites of swelling was similar between inpatients and specialist services with the majority experiencing edema of the leg or foot. More patients were diagnosed with primary lymphedema in the specialist services compared with inpatients, who also experienced a greater proportion with cancer-related secondary disease.

**FIG. 1.**
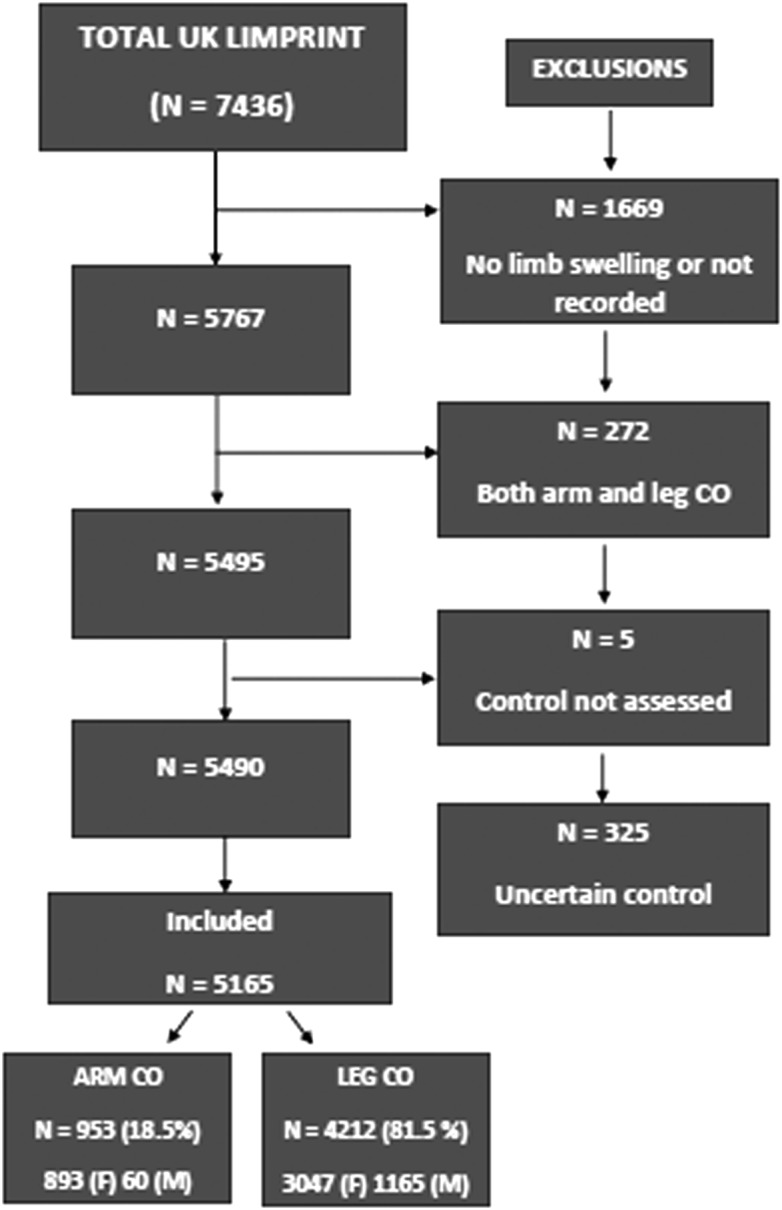
Study flow of participants.

In total 5767 patients with CO of one or more limbs were entered into the study of factors associated with control of swelling. Patients with both arm and leg swelling were excluded from this analysis (*n* = 272). Of the remainder 5495 were assessed for control of swelling. Of these, 325 were excluded on the basis that the professional was either unsure or did not know whether the swelling was controlled or not. The total number of patients analyzed was 5165, of which 1430 (27.7%) were assessed by their health professional as having poor control with 3735 (72.3%) having good control (Fig. 1). Of the total 3938 (76.3%) were women with a mean (SD) age of 65.3 (15.9) years. The 1225 men had a mean (SD) age of 64.9 (15.8) years. For the analysis, patients were divided into 953 (18.5%) with arm edema and 4212 (81.5%) with leg edema and were analyzed separately.

## Control of Arm Swelling

Table 2 gives demographic and mobility information on the 953 patients with arm edema. There was some evidence that men were less likely to have control over their CO than for women in this group. Those over 45 years had better control than those under, although there was some evidence that the very elderly group (>85 years) may have experienced lower control (OR = 0.77), although the confidence intervals were wide for this group (0.31, 1.88).

**Table 2.** Control of Arm Swelling: Demographic and Mobility Problems (*n* = 953)

	*No control*		*Control*			
	*N*	%	*N*	%	*OR 95%CI*	p*-value*
Gender						
Female	164	90.61	729	94.43	1.00	
Male	17	9.39	43	5.57	0.57 (0.32–1.02)	0.060
Age						
<45 years	12	6.67	39	5.05	1.00	
45–64 years	92	51.11	322	41.71	1.08 (0.54–2.14)	0.010
65–74 years	34	18.89	215	27.85	1.95 (0.93–4.08)	
75–84 years	28	15.56	161	20.85	1.77 (0.83–3.79)	
85+ years	14	7.78	35	4.53	0.77 (0.31–1.88)	
Obesity						
Normal weight	83	45.86	423	54.86	1.00	
Underweight	6	3.31	9	1.17	0.29 (0.10–0.85)	0.021
Obese	75	41.44	298	38.65	0.78 (0.55–1.10)	
Morbidly obese	17	9.39	41	5.32	0.47 (0.26–0.87)	
Arm mobility						
Full range of movement	128	70.72	561	72.67	1.00	
Limited range of movement	53	29.28	209	27.07	0.90 (0.63–1.29)	0.56
No function	0	0	2	0.26	—	

Control of CO was strongly associated with the levels of obesity, with higher BMI associated with poorer control. Underweight patients also appeared to have lower control, although this was based on few patients (*n* = 15) compared with those with normal weight. Limited range of arm movement had little impact on control compared with patients with full range of movement.

Table 3 examines the relationship between comorbidities and control of CO. Strong associations with control of CO were found with the presence of neurological disease and an acute infection (cellulitis) in the previous year. Other associations were present but did not achieve statistical significance. These may be, in part, due to small numbers of patients with these conditions.

**Table 3.** Control of Arm Swelling: Comorbidities (*n* = 953)

	*No control*		*Control*			
	N	*%*	N	*%*	*OR 95%CI*	p*-value*
Diabetes						
Absent	158	87.29	698	90.41	1.00	
Present	23	12.71	74	9.59	0.73 (0.44–1.20)	0.21
Heart failure/CHD						
Absent	168	92.82	735	95.21	1.00	
Present	13	7.18	37	4.79	0.65 (0.34–1.25)	0.20
Neurological disease						
Absent	1702	93.92	753	97.54	1.00	
Present	11	6.08	19	2.46	0.39 (0.18–0.83)	0.015
Peripheral arterial disease						
Absent	179	98.90	770	99.74		
Present	2	1.10	2	0.26		0.17^*^
Wound present						
Absent	174	96.13	757	98.06	1.00	
Present	7	3.87	15	1.94	0.53 (0.22–1.31)	0.16
History of cellulitis						
Absent	120	66.30	563	72.93	1.00	
Present	61	33.70	209	27.07	0.73 (0.52–1.03)	0.076
Cellulitis in past year						
Absent	148	81.77	718	93.01	1.00	
Present	33	18.23	54	6.99	0.34 (0.21–0.54)	<0.001

^*^ Fisher's exact test.

Details of the swelling are given in Table 4. While there was a large difference between the levels of control and swelling duration, it appears that this difference is stable over the patients whose CO was present for more than one year. In the subgroup with secondary cause (*n* = 930) CO control was more likely in the patients with cancer as a cause of their swelling but not for those with metastatic disease. The small number of patients with other secondary causes of CO meant that little could be gleaned from these analyses.

**Table 4.** Control of Arm Swelling: Details and Classification of Swelling (*n* = 953)

	*No Control*		*Control*			
	N	*%*	N	*%*	*OR 95%CI*	p*-value*
Swelling duration (*n* = 951)						
<1 year	42	23.20	51	6.62	1.00	
1–2 years	23	12.71	125	16.23	4.48 (2.45–8.19)	<0.001
2–5 years	42	23.20	206	26.75	4.04 (2.39–6.84)	
5–10 years	44	24.31	215	27.92	4.02 (2.39–6.78)	
>10 years	30	16.57	173	22.47	4.75 (2.70–8.34)	
Classification (*n* = 950)						
Primary	5	2.76	9	1.17	1.00	
Secondary	176	97.24	760	98.83	2.40 (0.79–7.25)	0.12
Secondary (*n* = 930)						
Cancer	157	89.71	710	94.04	1.00	
Noncancer	18	10.29	45	5.96	0.55 (0.31–0.98)	0.043
Cancer (*n* = 867)						
Cancer treatment						
Absent	16	10.19	20	2.82	1.00	
Present	141	89.81	690	97.18	3.91 (1.98–7.74)	<0.001
Cancer metastatic						
Absent	140	89.17	684	96.34	1.00	
Present	17	10.83	26	3.66	0.31 (0.17–0.59)	<0.001
Noncancer (*n* = 63)						
Venous						
Absent	17	94.44	39	86.67		
Present	1	5.56	6	13.33		0.66^*^
Immobility						
Absent	16	88.89	41	91.11		
Present	2	11.11	4	8.89		0.99^*^
Obesity						
Absent	17	94.44	45	100.0		
Present	1	5.56	0	0		0.29^*^
Other						
Absent	3	16.67	6	13.33		
Present	15	83.33	40	86.67		0.71^*^

^*^ Fisher's exact test.

Table 5 shows the associations between treatments and control. There was good evidence that skin care advice and use of compression garments were associated with better control, with little evidence of an association with antibiotic usage or massage. Multilayer bandaging was associated with poorer control of the swelling.

**Table 5.** Control of Arm Swelling: Previous and Current Treatments (*n* = 953)

	*No control*		*Control*			
	N	*%*	N	*%*	*OR 95%CI*	p*-value*
Massage						
Absent	124	68.51	521	67.49	1.00	
Present	57	31.49	251	32.51	1.05 (0.741–1.48)	0.79
Skin care advice						
Absent	28	15.47	33	4.27	1.00	
Present	153	84.53	739	95.73	4.10 (2.41–6.98)	<0.001
Compression garment						
Absent	21	11.60	54	6.99	1.00	
Present	160	88.40	719	93.01	1.75 (1.02–2.97)	0.040
Multilayer bandaging						
Absent	146	80.66	727	94.17	1.00	
Present	35	19.34	45	5.83	0.26 (0.16–0.42)	<0.001
Antibiotics						
Absent	169	93.37	741	95.98	1.00	
Present	12	6.63	31	4.02	0.59 (0.30–1.17)	0.13

## Control of Leg Swelling

Table 6 gives information on the control of leg swelling in relation to demographic details. While the difference between genders was less than for arm edema (OR = 0.84 vs. 0.57), the larger sample size achieved a standard level of statistical difference (*p* = 0.017). The observations on age were contradictory to those with arm edema. Patients over the age of 45 were less likely to have control of their swelling, although again there appeared to be little difference between the older age groups. Patients who were either underweight or overweight had better control of their swelling than those of normal weight, although this only achieved a statistical difference in the underweight and morbidly obese patients. Mobility was an important factor in control of swelling with strong association between poor mobility and failure to control the swelling. All comorbidities listed in Table 7 were strongly related to a lack of edema control.

**Table 6.** Control of Leg Swelling: Demographic and Mobility Problems (*N* = 4212)

	*No control*		*Control*			
	N	*%*	N	*%*	*OR 95%CI*	p*-value*
Gender						
Female	872	69.82	2175	73.41	1.00	
Male	377	30.18	788	26.59	0.84 (0.72–0.97)	0.017
Age						
<45 years	113	9.05	373	12.59	1.00	
45–64 years	376	30.13	961	32.43	0.77 (0.61–0.99)	
65–74 years	283	22.68	720	24.30	0.77 (0.60–0.99)	<0.001
75–84 years	312	25.00	639	21.57	0.62 (0.48–0.80)	
85+ years	164	13.14	270	9.11	0.50 (0.37–0.66)	
Obesity						
Normal weight	329	26.34	898	30.31	1.00	
Underweight	24	1.92	35	1.18	0.53 (0.31–0.91)	
Obese	488	39.07	1186	40.03	0.89 (0.76–1.05)	0.004
Morbidly obese	408	32.67	844	28.48	0.76 (0.64–0.90)	
Leg mobility						
Walks unaided	445	35.63	1648	55.62	1.00	
Walks with aid	589	47.16	1038	35.03	0.48 (0.41–0.55)	<0.001
Chair bound	187	14.97	264	8.91	0.38 (0.31–0.47)	
Bedbound	28	2.24	13	0.44	0.13 (0.06–0.24)	

**Table 7.** Control of Leg Swelling: Comorbidities (*n* = 4212)

	*No control*		*Control*			
	N	*%*	N	*%*	*OR 95%CI*	p*-value*
Diabetes						
Absent	963	77.10	2432	82.08	1.00	
Present	286	22.90	531	17.92	0.74 (0.63–0.86)	<0.001
Heart failure/CHD						
Absent	992	79.42	2586	87.28	1.00	
Present	257	20.58	377	12.72	0.56 (0.47–0.67)	<0.001
Neurological disease						
Absent	1107	88.63	2708	91.39	1.00	
Present	142	11.37	255	8.61	0.73 (0.59–0.91)	0.005
Peripheral arterial disease						
Absent	1223	97.92	2935	99.06	1.00	
Present	26	2.08	28	0.94	0.45 (0.26–0.77)	0.004
Wound						
Absent	950	76.06	2772	93.55	1.00	
Present	299	23.94	191	6.45	0.22 (0.18–0.27)	<0.001
History of cellulitis						
Absent	660	52.88	1700	57.39	1.00	
Present	588	47.12	1262	42.61	0.83 (0.73–0.95)	0.007
Cellulitis in past year						
Absent	959	76.78	2652	89.50	1.00	
Present	290	23.22	311	10.50	0.39 (0.33–0.46)	<0.001

There was a strong positive relationship between swelling duration and control (Table 8), which was similar to arm edema. Secondary edema was associated with poorer control, and noncancer edema was related to poorer control although with a lack of statistical significance. Swelling associated with cancer treatment as the cause of swelling was strongly associated with good control, whereas metastatic cancer was associated with poor control. Venous disease as a cause of swelling was associated with good control, while immobility and obesity causes were both strongly related to poor control.

**Table 8.** Control of Leg Swelling: Classification of Swelling (*n* = 4212)

	*No control*		*Control*			
	N	*%*	N	*%*	*OR 95%CI*	p*-value*
Swelling duration						
<1 year	154	12.34	78	2.63	1.00	
1–2 years	129	10.34	223	7.53	3.41 (2.41–4.83)	
2–5 years	260	20.83	624	21.07	4.74 (3.48–6.45)	<0.001
5–10 years	281	22.52	859	29.00	6.04 (4.45–8.18)	
>10 years	424	33.97	1178	39.77	5.49 (4.09–7.36)	
Classification						
Primary	118	9.54	541	18.53	1.00	
Secondary	1119	90.46	2379	81.47	0.46 (0.38–0.57)	<0.001
Secondary cause *n* = 3492						
Cancer	142	12.69	342	14.41	1.00	0.17
Noncancer	977	87.31	2031	85.59	0.86 (0.70–1.06)	
Cancer (*n* = 484)						
Cancer treatment						
Absent	45	31.69	28	8.19	1.00	
Present	97	68.31	314	91.81	5.20 (3.08–8.78)	<0.001
Cancer metastatic						
Absent	95	66.90	314	91.81	1.00	
Present	47	33.10	28	8.19	0.18 (0.11–0.30)	<0.001
Noncancer (*n* = 2925)						
Venous						
Absent	591	60.49	1149	56.57	1.00	
Present	386	39.51	882	43.43	1.18 (1.01–1.37)	0.042
Immobility						
Absent	482	49.33	1248	61.45	1.00	
Present	495	50.67	783	38.55	0.61 (0.52–0.71)	<0.001
Obesity						
Absent	601	61.51	1379	67.90	1.00	
Present	376	38.49	652	32.10	0.76 (0.64–0.89)	<0.001
Lymphatic filariasis						
Absent	973	99.59	2029	99.90		0.09^*^
Present	4	0.41	2	0.10		
Other						
Absent	440	45.04	799	39.34	1.00	0.001
Present	537	54.96	1232	60.66	1.26 (1.08–1.47)	

^*^ Fisher's exact test.

Table 9 gives the use of treatments in relation to control of swelling. Skin care advice, massage, and the use of compression garments were associated with good control of the edema. The use of multilayer bandaging and antibiotic usage were both related to poor control.

**Table 9.** Control of Leg Swelling: Previous and Current Treatments (*n* = 4212)

	*No control*		*Control*			
	N	*%*	N	*%*	*OR 95%CI*	p*-value*
Massage						
Absent	1120	89.67	2494	84.17	1.00	
Present	129	10.33	469	15.83	1.63 (1.33–2.01)	<0.001
Skin care						
Absent	346	27.70	171	5.77	1.00	
Present	903	72.30	2792	94.23	6.26 (5.13–7.63)	<0.001
Compression garment						
Absent	572	45.80	361	12.18	1.00	
Present	677	54.20	2602	87.82	6.09 (5.21–7.12)	<0.001
Multilayer bandaging						
Absent	969	77.58	2659	89.74	1.00	
Present	280	22.42	304	10.26	0.40 (0.33–0.47)	<0.001
Antibiotics						
Absent	1141	91.35	2840	95.85	1.00	
Present	108	8.65	123	4.15	0.46 (0.35–0.60)	<0.001

## Multivariable Analysis

Table 10 gives the multivariable analysis of the intrinsic factors in the control of swelling in patients with arm swelling. Factors identified for poor control were high levels of obesity, presence of neurological disease, recent infection, and short duration of CO. For patients with leg disease the independent factors were high levels of obesity, poor mobility, presence of heart disease, presence of a wound, recent infection, and short duration of CO (Table 11).

**Table 10.** Independent Factors Associated with Control of Arm Swelling

	*Odds ratio*	*95% CI*	*z-score*	p*-value*
Obesity				
Normal	1.00			
Underweight	0.28	0.09–0.86	−2.42	0.015
Obese	0.73	0.51–1.06		
Morbidly obese	0.45	0.24–086		
Neurological disease				
Absent	1.00			
Present	0.32	0.14–0.70	−2.83	0.005
Infection within past year				
Absent	1.00			
Present	0.31	0.19–0.50	−4.71	<0.001
Swelling duration				
<1 year	1.00	1.00		
1–2 years	4.96	2.66–9.26		
2–5 years	4.20	2.44–7.24	4.49	<0.001
5–10 years	4.60	2.66–7.92		
>10 years	5.11	2.85–9.14		

**Table 11.** Independent Factors Associated with Control of Leg Swelling

	*Odds ratio*	*95% CI*	*z-score*	p*-value*
Obesity				
Normal	1.00			
Underweight	1.07	0.56–2.03	−3.39	0.001
Obese	0.78	0.65–0.94		
Morbidly obese	0.69	0.57–0.84		
Mobility				
Walks unaided	1.00			
Walks with aid	0.56	0.48–0.66	−8.85	<0.001
Chair bound	0.45	0.36–0.56		
Bedbound	0.19	0.09–0.40		
Heart disease				
Absent	1.00			
Present	0.74	0.61–0.89	−3.09	0.002
Wound present				
Absent	1.00			
Present	0.28	0.23–0.35	−11.86	<0.001
Infection within past year				
Absent	1.00			
Present	0.47	0.38–0.56	−7.81	<0.001
Swelling duration				
<1 year	1.00	1.00		
1–2 years	3.06	2.12–4.42		
2–5 years	4.83	3.48–6.71	9.64	<0.001
5–10 years	6.33	4.57–8.77		
>10 years	5.52	4.03–7.57		

## Discussion

Findings from this study would indicate that understanding the subjective control of swelling based on professional assessment is complex. Many different sociodemographic, clinical, and treatment issues influence control. The distribution of the site of swelling is important and indicates that leg edema is associated with a worsening prognosis with increasing age that is aggravated significantly by the level of obesity and immobility. Cellulitis has emerged as an important factor in many of the LIMPRINT studies. In this analysis an episode of cellulitis in the last year was found to be related to poor control.^9^

The results from the earlier study that assessed subjective control of swelling were supported in this analysis and would indicate that even within specialist services that are providing CDT control of CO may still be difficult to achieve and maintain due to the complexities of the patient population.^10^ It is therefore likely that control of swelling will be worse within general health services with poor access to specialist services. Previous research has shown that control of swelling over time is not maintained after CDT and that there is a poorer response to those who require repeated episodes of CDT.^11^

Caution over the link to treatment and control of swelling in this study should be made. While compression hosiery was associated with better control than multilayer bandaging this may simply be a reflection that new patients are requiring CDT and have a more severe presentation of CO rather than those who are self-managing with compression hosiery during the maintenance phase of treatment.

## Limitations

Several limitations must be acknowledged within the study. As previously discussed, objective descriptions and methods to define control of swelling do not currently exist.^12,13^ Every attempt was made to standardize and reduce the number of people collecting data within this study and to seek clarification over professional judgment of control; however, these will always be subject to bias in the absence of clear definitions and correlation against other objective measures.

Despite this, the sample size is large, and every attempt was made to reduce the natural biases that may exist. Data were excluded from all participants where the decision about control of swelling was uncertain and access to specialized Lymphedema teams assisted in increasing the accuracy of the decisions that were made.

A cross-sectional study is also limited to an assessment at one time point, and longitudinal studies are required to examine the natural history of this phenomena. Further studies in more general populations would also help to define this important area more clearly and to correlate these with robust clinical and other psychosocial outcomes.

## Conclusion

Professional judgment of control of CO in arm and leg CO is complex. To avoid double counting, patients with both arm and leg CO were excluded (N=272). A total of 5165 patients were included in the study with exclusions for: no limb swelling or not recorded (1669), having concurrent arm/leg CO (272), control of assessment missing (5) and professional being unsure of control status of CO (325).

Arm swelling occurred in 953 (18.5%) with leg CO in 4212 (81.5%). Poor control was found in 1430 (27.2%) and good control in 3735 (72.3%). Control of arm swelling was worse in men and control increased overall in those aged over 45 years. In contrast control of CO worsened in those with leg CO with increasing age and multiple co-morbidities. Obesity and cellulitis, particularly an episode in the last year were associated with poor control. Independent risk factors for arm CO were, obesity, neurological disease and cellulitis in the last year and for leg CO, obesity, poor mobility, heart disease, presence of a wound, cellulitis in the last year and duration of swelling.

This study highlights the complexity of patients with CO and the challenges associated with obtaining control of CO within specialist services. The research highlights the importance of addressing the complex issues associated with lower limb swelling and the importance of issues such as obesity and immobility which are more challenging to influence. Risk factor analysis within this study will help the prioritization of treatment for high risk patient groups who may require more intensive therapy to standardize their condition. Research to improve on measurement of outcomes is urgently needed.
